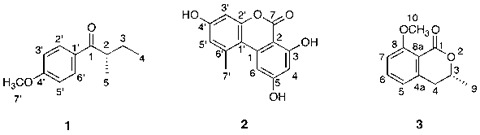# Induced biosyntheses of a novel butyrophenone and two aromatic polyketides in the plant pathogen *Stagonospora nodorum*

**DOI:** 10.1007/s13659-013-0055-2

**Published:** 2013-08-17

**Authors:** Xiao-Long Yang, Takayoshi Awakawa, Toshiyuki Wakimoto, Ikuro Abe

**Affiliations:** 1 Graduate School of Pharmaceutical Sciences, The University of Tokyo, 7-3-1 Hongo, Bunkyo-ku, Tokyo, 113-0033 Japan; 2 College of Pharmaceutical Science, Hebei University, Baoding, 071002 China

**Keywords:** *Stagonospora nodorum*, butyrophenone, polyketides, epigenetic manipulation

## Abstract

Fungal aromatic compounds comprise an important and structurally diverse group of secondary metabolites. Several genome sequencing projects revealed many putative biosynthetic gene clusters of fungal aromatic compounds, but many of these genes seem to be silent under typical laboratory culture conditions. To gain access to this untapped reservoir of natural products, we utilized chemical epigenetic modifiers to induce the expression of dormant biosynthetic genes. As a result, the concomitant supplementation of the histone deacetylase inhibitors suberoylanilide hydroxamic acid (500 μM) and nicotinamide (50 μM) to the culture medium of a fungal pathogen, *Stagonospora nodorum*, resulted in the isolation of three aromatic compounds (**1–3**), including a novel natural butyrophenone, (+)-4′-methoxy-(2*S*)-methylbutyrophenone (**1**), and two known polyketides, alternariol (**2**) and (−)-(3*R*)-mellein methyl ether (**3**).

## Introduction

Fungal aromatic compounds are structurally and functionally diverse, as exemplified by the mycotoxin aflatoxin, the pigment emodin, and the antifungal drug griseofulvin.[1–3] Some of them are produced by polyketide synthases (PKSs), and have been linked to their biosynthetic genes. However, there are a large number of PKS genes with products that have not been identified in several fungal genome sequences, indicating that numerous cryptic biosynthetic pathways of natural products remain to be discovered. *Stagonospora nodorum* is a destructive wheat pathogen.[4] It belongs to *Dothideomycetes*, a class of filamentous fungi that includes many plant pathogens, and thus important as a model for pathology study. Previous studies aiming toward the comprehension of plant diseases demonstrated the critical roles of host-specific proteinaceous toxins and various primary metabolic pathways for effective fungal pathogenicity.[5–9] Nevertheless, only a few papers regarding the capacity of this strain to produce secondary metabolites have been published, except for one mycotoxin, alternariol.[8] Recently, the whole genome sequence of *S. nodorum* revealed that it contains 19 genes encoding PKSs, 8 genes encoding non-ribosomal peptide synthetases (NRPSs), and one gene encoding a PKS-NRPS hybrid, to produce cryptic natural products that outnumber the currently known metabolites from this fungus.[10] However, many of these putative biosynthetic genes seem to be silent under a variety of laboratory culturing conditions. To trigger the expression of these gene clusters, an effective method is to use epigenetic modifying agents, including histone deacetylase (HDAC) and DNA methyltransferase inhibitors, for manipulating the fungal epigenome.[11] In our study, screening of the secondary metabolites produced by *S. nodorum*, grown in the presence of these inhibitors, revealed that the concomitant supplementation of the HDAC inhibitors to the culture medium induced significant changes in the production of metabolites. This finding led to the isolation of a novel natural butyrophenone, (+)-4'-methoxy-(2*S*)-methylbutyrophenone (**1**), along with two known aromatic polyketides, alternariol (**2**) and (–)-(3*R*)-mellein methyl ether (**3**). Details of the isolation and structural elucidation of **1**–**3** are reported herein.

## Results and Discussion

*S. nodorum* FGSC 10173 was incubated in 100 mL of potato dextrose broth (PDB), in the presence and absence of 500 μM SBHA and 50 μM nicotinamide. The ethyl acetate extract of the culture filtrate was analyzed by high performance liquid chromatography (HPLC). As a result, the production of two new products (**1** and **3**) was induced, and the intensity of one peak for compound **2** was enhanced (Figure 1).

Compound **1** was obtained as a white solid. Its molecular formula was established as $C_{12}H_{16}O_2$, by a pseudomolecular ion peak at *m/z* 193.1234 $[M + H]^+$ (calcd for $C_{12}H_{17}O_2$, 193.1223) in the positive HRESIMS, with 5 degrees of unsaturation. The $^1$H NMR analysis revealed the presence of a 1,4-disubstituted aromatic ring ($\delta_H$ 7.71 (2H, d, $J$ = 8.5 Hz),

*To whom correspondence should be addressed. E-mail: abei@mol.f.u-tokyo.ac.jp

**Figure 1.** HPLC analyses of culture broths of *S. nodorum*: (A) Incubated in PDB, (B) Incubated in PDB containing SBHA (500 μM) + nicotinamide (50 μM). Elutions were monitored by UV absorption at 254 nm.

6.80 (2H, d, $J$ = 8.5 Hz)), three methyl groups ($\delta_H$ 3.85 (3H, s), 1.25 (3H, d, $J$ = 6.5 Hz), 0.89 (3H, t, $J$ = 7.5 Hz)), one methine group ($\delta_H$ 3.15 (1H, m)), and one methylene group ($\delta_H$ 1.60 (1H, m), 1.85 (1H, m)). Analyses of its $^{13}$C NMR, HSQC and $^1$H-$^1$H COSY data further revealed the presence of a 1,4-disubstituted aromatic ring ($\delta_C$ 128.0 (s, C-1'), 132.7 (d, C-2', and 6'), 114.9 (d, C-3', and 5'), 163.3 (s, C-4')), one methoxy carbon ($\delta_C$ 52.9 (q, C-7')), and a 2-methylbutan-1-one moiety ($\delta_C$ 191.5 (s, C-1), 36.5 (d, C-2), 27.8 (t, C-3) 11.0 (q, C-4), 17.4 (q, C-5)). Further detailed interpretation of the HMBC correlations from H-2' and H-6' to C-1, and from H-7' to C-4', allowed us to assign the methoxy group and the 2-methylbutan-1-one moiety to C-4' and C-1', respectively. Finally, compound **1** was identified as (+)-4'-methoxy-(2*S*)-methylbutyrophenone (Figure 2), which was previously reported as a synthetic compound.[12] The absolute configuration of C-2 was determined to be *S*, by comparing its optical rotation to the reported value.[12]

Compound **2** was obtained as a white solid. Its molecular formula was established as $C_{14}H_{10}O_5$, by a pseudomolecular ion peak at *m/z* 259.0612 [M + H]$^+$ (calcd for $C_{14}H_{11}O_5$, 259.0601) in the positive HRESIMS. Analyses of its $^{13}$C NMR and HSQC data revealed 14 carbon resonances, including one ester carbonyl carbon, four aromatic methine carbons, eight aromatic quaternary carbons, and one methyl carbon. The $^1$H NMR spectrum also displayed four $sp^2$ methines ($\delta_H$ 6.34 (1H, d, $J$ = 1.7 Hz, H-4), 7.21 (1H, d, $J$ = 1.7 Hz, H-6), 6.61 (1H, d, $J$ = 2.3 Hz, H-3'), 6.69 (1H, d, $J$ = 2.3 Hz, H-5')), featuring two 1,2,3,5-tetrasubstituted aromatic rings deduced from their coupling constants, and one methyl ($\delta_H$ 2.68 (3H, s, H-7')). Thus, its NMR data were identical to those of alternariol, a known mycotoxin metabolite from the mold *Alternaria tenuis*.[13]

Compound **3** was obtained as a white solid. Its molecular formula was established as $C_{11}H_{12}O_3$, by a pseudo-molecular ion peak at *m/z* 193.0837 [M + H]$^+$ (calcd for $C_{11}H_{13}O_3$, 193.0859) in the positive HRESIMS. Analysis of the $^1$H NMR spectrum revealed the presence of 1,2,3-trisubstituted aromatic ring signals ($\delta_H$ 6.85 (1H, d, $J$ = 7.5 Hz, H-5), 7.47 (1H, dd, $J$ = 8.5, 7.5 Hz, H-6), 7.00 (1H, d, $J$ = 8.5 Hz, H-7)), one oxygenated methine ($\delta_H$ 4.45 (1H, m, H-3)), one methylene ($\delta_H$ 2.87 (2H, m, H-4)), and two methyls ($\delta_H$ 1.31 (3H, d, $J$ = 7.0 Hz, H-9), 3.77 (3H, s, H-10)). The $^{13}$C NMR spectrum revealed 11 carbon signals, due to one ester carbonyl, three aromatic methine carbons, three aromatic quaternary carbons, one oxygenated methine carbon, one methylene carbon, and two methyl carbons. A comparison of the NMR data and the optical rotation value revealed that **3** is identical to (–)-(3*R*)-mellein methyl ether, previously isolated from the mangrove-derived fungus *Xylaria cubensis* (Figure 2).[14]

Compounds **1**–**3** were tested for their antifungal activities against three fungal strains, including *Fusarium solani*, *Aspergillus terreus* and *Candida albicans*.[15] The results showed that these three compounds displayed moderate activities against the three tested fungal strains (Table 1).

Although *S. nodorum* contains 19 genes encoding PKSs, few polyketides were discovered from this fungus. Interestingly, these PKS gene clusters are dormant under normal culture conditions, but the concomitant supplementation of the HDAC inhibitors SBHA (500 μM) and nicotinamide (50 μM) to the culture medium of *S. nodorum* facilitated the production of three aromatic compounds (**1**–**3**). Upon further

**1** **2** **3**

**Figure 2.** The structures of compounds **1–3**

**Table 1. Antifungal effect of compounds 1–3**

| | MIC (μg/mL) | | | |
|---|---|---|---|---|
| **Fungal strain** | **1** | **2** | **3** | ketoconazole |
| *Fusarium solani* | 50 | 10 | 100 | > 100 |
| *Aspergillus terreus* | 50 | 50 | 50 | 25 |
| *Candida albicans* | > 100 | 50 | > 100 | 3.1 |

isolation, we identified the novel natural butyrophenone (**1**), together with two known aromatic polyketides (**2** and **3**). Our findings confirmed that chemical epigenetic manipulation is an effective strategy to access the biosynthetic potential of this fungus.

The novel (+)-4'-methoxy-(2*S*)-methylbutyrophenone (**1**) could be derived from the polyketide biosynthetic pathway. A related compound, 1-(2,4,6-trihydroxyphenyl)butan-1-one, was isolated from the endophytic fungus *Paeonia delavayi*,[16] which led us to assume that the phloroglucinol precursor is possibly converted into the para-substituted phenol scaffold of **1** by reductases. Furthermore, the (2*S*)-methylbutyryl moiety of **1** could be generated by a diketide-producing PKS, as in the case of the diketide side chain formation by LovF in lovastatin biosynthesis,[17] and then transferred to another PKS that catalyzes three chain extensions and a Dieckmann condensation, to produce the phloroglucinol scaffold. In fact, we found *SNOG_09623*, encoding a PKS with the same domain organization as those of LovF, and *SNOG_09622*, encoding a type III PKS, in the *S. nodorum* genome. The two genes are clustered in the chromosome, but without any methyltransferase gene nearby. The polyketide chain transfer between two PKSs was reported for the bacterial PKS system of the alkylresorcinol and alkylpyrone biosynthesis in *Azotobacter vinelandii*[18], and for the formation of phlorocaprophenone in *Dictyostellium discoidium* (type III PKS Steely 2).[19]

On the other hand, although (–)-(3*R*)-mellein methyl ether (**3**) is widely distributed in fungi, its biosynthetic enzymes have not been reported. Recently, SACE5532 from the actinobacterium, *Saccharopolyspora erythraea*, which consists of ketosynthase (KS), acyltransferase (AT), thioester hydrolase (TH), and ketoreductase (KR) domains, was identified as a PKS that produces (*R*)-(–)-mellein.[20] Therefore, a PKS with identical domains to those of SACE5532 in *S. nodorum* should be a candidate for a mellein PKS. Two PKS genes exist in *S. nodorum*, *SNOG_00477* and *SNOG_14927*, and they encode PKSs with identical domain structures to those of SACE5532. Thus, one of them is likely to be the PKS that generates mellein. This information illustrates the application of chemical epigenetic manipulation to a genome-sequenced strain, to provide helpful information toward identifying biosynthetic genes.

## Experimental Section

**HPLC Analysis of the Compounds Accumulated in the Broth of *S. nodorum*.** *S. nodorum* FGSC 10173 was obtained from the Fungal Genetic Stock Center. The fungal strain was cultured on potato dextrose agar (PDA) at 28 °C for 7 days, and then inoculated into 100 mL of PDB containing 500 μM SBHA and/or 50 μM nicotinamide in 500 mL Erlenmeyer flasks, under shaking conditions at 28 ºC for 7 days. The culture broths were extracted three times with ethyl acetate. After evaporation *in vacuo*, the crude extracts were subjected to an HPLC analysis. The HPLC analysis was performed at a flow rate of 0.8 mL/min on an ODS column (Cosmosil 5C18 PAQ Waters, 4.6 × 250 mm), with a mixture of $H_2O$ and $CH_3CN$ (0–30 min, 10–100% $CH_3CN$; 30–50 min, 100% $CH_3CN$; 50–55 min, 100–10% $CH_3CN$).

**Isolation of Compounds (1–3).** To isolate the induced compounds, *S. nodorum* was inoculated in 20 × 100 mL of PDB, containing 500 μM SBHA and 50 μM nicotinamide, in 500 mL Erlenmeyer flasks under shaking conditions at 28 ºC for 7 days. The culture broths were extracted three times with ethyl acetate. After evaporation *in vacuo*, the crude extract was directly separated by preparative HPLC (Shimadzu, LC-20AD and SPD-20A Prominence Diode Array Detector), using a Cosmosil 5C18-PAQ Waters column (10 × 250 mm, Nacalai Tesque, Kyoto) eluted with a mixture of $H_2O$ (solvent A) and $CH_3CN$ (solvent B), both containing 0.1% acetic acid, at a flow rate of 2.5 mL/min. The separation performance was equivalent to that of the analytical method, which led to the isolation of **1** (1.28 mg, $t_R$ = 21.1 min, 35% $CH_3CN$ over 15 min), **2** (2.28 mg, $t_R$ = 17.8 min, 35% $CH_3CN$ over 15 min), and **3** (6.96 mg, $t_R$ = 16.7 min, 35% $CH_3CN$ over 15 min).

**Antifungal Activity Assays.** Antifungal bioassays were conducted according to the previously reported procedure.[15] The fungal strains, *Fusarium solani*, *Aspergillus terreus* and *Candida albicans*, were grown on PDA. Targeted microbes (3–4 colonies) were prepared from broth cultures (28 ºC for

72 h), and the final fungal spore suspensions (in PDB) were $10^4$ mycelial fragments/mL. Test samples (10 mg/mL as a stock solution in DMSO and serial dilutions) were transferred to a 96-well clear plate in triplicate, and the suspension of the test organisms was added to each well, to achieve a final volume of 100 μL (ketoconazole was used as the positive control). After incubation, the minimum inhibitory concentration (MIC) was defined as the lowest test concentration that completely inhibited the growth of the test organisms.

**(+)-4'-Methoxy-(2*S*)-methylbutyrophenone (1):** white solid; $[\alpha]_D^{24}$ + 7.5 (*c* 0.07, MeOH); UV (MeOH) $\lambda_{max}$ (log $\varepsilon$) 265, 284, 315 nm; $^1$H NMR ($CD_3OD$, 500 MHz): $\delta$ 0.89 (3H, t, *J* = 7.5 Hz, H-4), 1.25 (3H, d, *J* = 6.5 Hz, H-5), 1.60 (1H, m, H-3a), 1.85 (1H, m, H-3b), 3.15 (1H, m, H-2), 3.85 (3H, s, H-7'), 6.80 (2H, d, *J* = 8.5 Hz, H-3' and 5'), 7.71 (2H, d, *J* = 8.5 Hz, H-2' and 6'); $^{13}$C NMR (125 MHz, $CD_3OD$): $\delta$ 191.5 (s, C-1), 163.3 (s, C-4'), 132.7 (d, C-2' and 6'), 128.0 (s, C-1'), 114.9 (d, C-3' and 5'), 52.9 (q, C-7'), 36.5 (d, C-2), 27.8 (t, C-3), 17.4 (q, C-5), 11.0 (q, C-4). ESIMS (positive) *m/z* 193 $[M + H]^+$; HRESIMS *m/z* 193.1234 (calcd for $C_{12}H_{17}O_2$, 193.1223).

**Alternariol (2):** white solid; UV (MeOH) $\lambda_{max}$ (log $\varepsilon$) 255, 297, 335 nm; $^1$H NMR (DMSO-$d_6$, 500 MHz): $\delta$ 2.68 (3H, s, H-7'), 6.34 (1H, d, *J* = 1.7 Hz, H-4), 6.61 (1H, d, *J* = 2.3 Hz, H-3'), 6.69 (1H, d, *J* = 2.3 Hz, H-5'), 7.21 (1H, d, *J* = 1.7 Hz, H-6); $^{13}$C NMR (125 MHz, DMSO-$d_6$): $\delta$ 166.3 (s, C-5), 165.2 (s, C-7), 164.6 (s, C-3), 159.0 (s, C-4'), 153.2 (s, C-2'), 138.8 (s, C-6'), 138.1 (s, C-1), 118.1 (d, C-5'), 109.5 (s, C-1'), 105.0 (d, C-6), 102.1 (d, C-3'), 101.5 (d, C-4), 97.8 (s, C-2), 25.8 (q, C-7'). ESIMS (positive) *m/z* 259 $[M + H]^+$. HRESIMS *m/z* 259.0612 (calcd for $C_{14}H_{11}O_5$, 259.0601).

**(–)-(3*R*)-Mellein methyl ether (3):** white solid; $[\alpha]_D^{24}$ – 324.0 (*c* 0.52, MeOH); UV (MeOH) $\lambda_{max}$ 243, 306 nm; $^1$H NMR (DMSO-$d_6$, 500 MHz): $\delta$ 1.31 (3H, d, *J* = 7.0 Hz, H-9), 2.87 (2H, m, H-4), 3.77 (3H, s, H-10), 4.45 (1H, m, H-3), 6.85 (1H, d, *J* = 7.5 Hz, H-5), 7.00 (1H, d, *J* = 8.5 Hz, H-7), 7.47 (1H, dd, *J* = 8.5, 7.5 Hz, H-6); $^{13}$C NMR (125 MHz, $CD_3OD$): $\delta$ 166.4 (s, C-1), 163.5 (s, C-8), 144.8 (s, C-4a), 137.3 (d, C-6), 121.6 (d, C-5), 115.1 (s, C-8a), 113.2 (d, C-7), 77.1 (d, C-3), 57.4 (q, C-10), 37.6 (t, C-4), 21.8 (q, C-9); ESIMS (positive) *m/z* 193 $[M + H]^+$; HRESIMS *m/z* 193.0837 (calcd for $C_{11}H_{13}O_3$, 193.0859).

## Acknowledgments

This work was financially supported by Grants-in-Aid for Scientific Research from the Ministry of Education, Culture, Sports, Science and Technology, Japan, and by programs from the National Natural Science Foundation Province of China (21202033), the Natural Science Foundation of Hebei (C2012201047), and the Foundation of Hebei University (179).

**Open Access** This article is distributed under the terms of the Creative Commons Attribution License which permits any use, distribution, and reproduction in any medium, provided the original author(s) and source are credited.
